# Glutamine as sole nitrogen source prevents induction of nitrate transporter gene *NRT2.4* and affects amino acid metabolism in Arabidopsis

**DOI:** 10.3389/fpls.2024.1369543

**Published:** 2024-03-25

**Authors:** Nataliia Svietlova, Liza Zhyr, Michael Reichelt, Veit Grabe, Axel Mithöfer

**Affiliations:** ^1^ Research Group Plant Defense Physiology, Max Planck Institute for Chemical Ecology, Jena, Germany; ^2^ Department of Biochemistry, Max Planck Institute for Chemical Ecology, Jena, Germany; ^3^ Microscopic Imaging Service Group, Max Planck Institute for Chemical Ecology, Jena, Germany

**Keywords:** Arabidopsis, nitrogen-deficiency, nitrate, glutamine, amino acids, high-affinity nitrate transporters (NRTs), *NRT2.4*, *NRT2.5*

## Abstract

Plants assimilate inorganic nitrogen (N) to glutamine. Glutamine is the most abundant amino acid in most plant species, the N-supplying precursor of all N-containing compounds in the cell and the first organic nitrogen molecule formed from inorganic nitrogen taken up by the roots. In addition to its role in plant nutrition, glutamine most likely also has a function as a signaling molecule in the regulation of nitrogen metabolism. We investigated whether glutamine influences the high-affinity transporter system for nitrate uptake. Therefore, we analyzed the expression of the nitrate transporter *NRT2.4*, which is inducible by N deficiency, in *Arabidopsis thaliana* grown under different nitrogen starvation scenarios, comparing nitrate or glutamine as the sole nitrogen source. Using the reporter line *ProNRT2.4:GFP* and two independent knockout lines, *nrt2.4-1* and *nrt2.4-2*, we analyzed gene expression and amino acid profiles. We showed that the regulation of *NRT2.4* expression depends on available nitrogen in general, for example on glutamine as a nitrogen source, and not specifically on nitrate. In contrast to high nitrate concentrations, amino acid profiles changed to an accumulation of amino acids containing more than one nitrogen during growth in high glutamine concentrations, indicating a switch to nitrogen storage metabolism. Furthermore, we demonstrated that the *nrt2.4-2* line shows unexpected effects on *NRT2.5* gene expression and the amino acids profile in shoots under high glutamine supply conditions compared to Arabidopsis wild type and *nrt2.4-1*, suggesting non-*NRT2.4*-related metabolic consequences in this knockout line.

## 1 Introduction

Nitrogen (N) is an essential macronutrient for plant growth and productivity. Plants absorb N from the soil mainly in the inorganic form of nitrate (NO_3_^−^) and ammonium (NH_4_^+^). While the root can assimilate ammonium directly, nitrate is mostly first transported to the shoot. There nitrate is reduced to ammonium in various enzymatic steps, transferred to the amino acid glutamine by glutamine synthetase and further introduced into the metabolism by aminotransferases. In addition, plants also have the ability to absorb organic nitrogen from soil such as amino acids (AA), peptides, urea, and other nitrogen-containing compounds (Ortiz-Lopez et al., 2000; Yao et al., 2020). Andrews et al. (2013) concluded in a comprehensive review that the form of N, which is available to and taken up by plants can influence timing and rate of seed germination, leaf expansion and function, shoot-to-root dry matter partitioning, and root architecture. Amino acids, which ubiquitously occur in soils due to hydrolysis of soil proteins, are a well-available form of organic N (Näsholm et al., 2009). They can be an important source of N for plants and account for 10% but can go up to 40% of the total soluble N in the soil (Gioseffi et al., 2012). Especially in cropping systems that rely on the recycling and decomposition of organic N sources, AA can have a significant contribution to N-input and represent an available N-pool (El-Naggar et al., 2009). Therefore, this source of nitrogen for plant nutrition should not be underestimated.

In higher plants, inorganic (NH_4_^+^, NO_3_^−^) N uptake and distribution is mediated by transporters with high (HATS) and low (LATS) affinities (Wang et al., 2012; Hao et al., 2020). NO_3_^−^ transporters are proteins encoded by four gene families: *Nitrate Transporters 1 (NRT1s), nitrate Transporters 2 (NRT2s)*, *chloride channels* (*CLCs*) and *slow anion channel* associated homologues (*SLAC/SLAHs*) (Wang et al., 2012). The *NRT* gene families of nitrate transporters in Arabidopsis contain 53 *NRT1* and 7 *NRT2* members (Orsel et al., 2002; Okamoto et al., 2003; Vidal et al., 2020), six of which are involved in nitrate uptake by roots (NRT1.1, NRT1.2 and NRT2.1, NRT2.2, NRT2.4, NRT2.5) (Vidal et al., 2020). NRT1s and NRT2s nitrate transporter gene homologues were classified as nitrate-inducible, nitrate-repressible, or nitrate-constitutive (Vidal et al., 2020). *AtNRT1.1*, *2.1*, and *2.2* were strongly and transiently induced by NO_3_^–^. Influx studies indicated that *AtNRT1.1* and *AtNRT2.1* belong to the LATS and HATS, respectively (Okamoto et al., 2003). By contrast, *AtNRT2.4* showed only modest induction both in shoots and roots, while expression of *AtNRT2.5* was strongly suppressed by nitrate uptake in both roots and shoots. Actually, that means both genes were induced by NO_3_^–^ deficiency (Kiba et al., 2012; Lezhneva et al., 2014). Finally, *AtNRT1.2*, *1.4*, *2.3*, *2.6*, and *2.7*, are characterized by a constitutive expression pattern (Vidal et al., 2020).

In addition, plants express a variety of different amino acid transporters with overlapping specificities and affinities, many of which expressed in roots (Fischer et al., 1998). There are multiple families of amino acid transporters belonging to three major families: ATF (amino acid transporter family, also called AAP, amino acid permease family), APC (amino acid-polyamine-choline transporter family) and UMAMIT (usually multiple acids move in and out transporter family). Some of these transporters take part in uptake of amino acids from the soil, for example AAP1, APP3, AAP5, UMAMIT1, Proline Transporter 2 (ProT2), and Lysine Histidine Transporter 1 (LHT1) (Ortiz-Lopez et al., 2000; Tegeder, 2012; Dinkeloo et al., 2018; Yao et al., 2020). Noteworthy, there is a particular role for LHT1 in the uptake from soil and intracellular distribution of Gln (Svennerstam et al., 2007; Liu et al., 2010). An Arabidopsis *lht1* knock out mutant showed broad pathogen resistance due to Gln-deficiency in chloroplasts and salicylic acid accumulation demonstrating the importance of at least the amino acid Gln and its homeostasis for the plant in plant pathogen interactions (Liu et al., 2010).

The fundamental demand of AA in any organism needs tight AA metabolism to sustain physiological homeostasis. In plants, there are a number of indications that AA metabolism undergoes dynamic changes to control particular growth and development events (Kawade et al., 2023). A large number of studies have shown that also exogenous amino acids present in the underground environment or leaf surface can be taken up by plants, and can have strong impacts on plant growth and/or defense response (Han et al., 2022; and references therein). When added at high concentrations (≥ 1mM) to tobacco (*Nicotiana sylvestris*) cell cultures, amino acids have an inhibitory effect on plant growth, very likely due to feedback inhibition of specific biosynthetic pathways (Bonner et al., 1996; Bonner and Jensen, 1997). Every amino acid causes amino acid-mediated growth inhibition called general amino acid inhibition, with the exception of L-Glutamine (Gln). In fact, Gln completely overcomes general amino acid inhibition (Bonner et al., 1996; Bonner and Jensen, 1997). Aspartate (Asp) and some branched chain amino acids inhibited root growth in barley (*Hordeum vulgare*) (Rognes et al. 1986) and Glutamate (Glu) inhibited cell elongation in *Arabidopsis thaliana* (ecotype Columbia) roots (Sivaguru et al., 2003). However, out of all 20 proteinogenic amino acids, only Glu affects root growth in Arabidopsis (most in ecotype C24), when added singly (Walch-Liu et al., 2006). Related amino acids such as Asp, GABA or Gln did not induce growth inhibitory effects at the low concentrations (50 µM) that Glu was effective; even millimolar concentrations of Gln had no effect on root growth in Arabidopsis (Walch-Liu et al., 2006). At these high concentrations, foliar spray of Glycine (Gly) and Gln stimulate lettuce (*Lactuca sativa*) growth (Noroozlo et al., 2019). In a poplar (*Populus deltoides × P. euramericana*) hybrid (Nanlin895), it was demonstrated that Gln concentrations < 0.5 mM as the sole N source had positive effects on various physiological and growth parameters, while concentrations > 0.5 mM showed adverse effects (Han et al., 2022). A very recent study showed growth promoting effects of Asparagine (Asn) and Gln on *A. thaliana* leaves in the mM range (Lardos et al., 2024).

The ability to monitor the cellular N status is essential for maintaining metabolic homeostasis, growth, and development in plants (Xuan et al., 2017). Different N-sensory systems are discussed to fulfill this role and further signaling, eventually leading to appropriate physiological responses. These systems include the TOR (target of rapamycin) signaling pathway, the family of GLRs (glutamate-like receptors), the GCN2 (general control non-derepressible 2) pathway, and the plastidic PII-dependent pathway (Gent and Forde, 2017). All of those have in common that they are supposed to bind amino acids for their monitoring. Considering that Gln is the most abundant free amino acid in plants, the first organic acceptor of inorganic N, and the key N-providing compound for the synthesis of all N-containing compounds in the plant cell, Gln is a very likely candidate (Lee et al., 2023). Thus, the existence of a common Gln-sensing mechanism (P_II_) that is widely distributed in the plant kingdom, is not surprising (Chellamuthu et al., 2014; Lee et al., 2023). Strikingly, P_II_ is non-functional in Brassicaceae, including Arabidopsis (Chellamuthu et al., 2014). Therefore, it was reasonable and of major interest, to examine the role of exogenous Gln in Arabidopsis plants.

In combination with a submillimolar nitrate content to break dormancy and enable germination, Gln at mM concentrations was identified as by far the most efficient biostimulatory AA in Arabidopsis (Lardos et al., 2024). This study confirmed previous results that also showed that Gln promotes Arabidopsis growth (Forsum et al., 2008). Here, we investigated the effects of low and high levels of exogenous Gln as the sole N source without additional nitrate. Since the main nitrogen source for the plant is inorganic nitrate, we investigated whether the high-affinity nitrate transporters (*NRT2.4* and *NRT2.5*) are induced even when Gln is available as nitrogen source. Under these nutritional conditions, amino acid metabolism, i.e. their different levels, was also analyzed in comparison to nitrate as N source. We could show that the expression of *NRTs* depends more on available nitrogen in general than on nitrate in particular. In contrast to high nitrate concentrations, the amino acid profile in shoots and roots changed significantly at high Gln supply. Furthermore, we demonstrate that the Arabidopsis line *nrt2.4-2* has side effects on *NRT2.5* gene expression and shoot amino acid profiles.

## 2 Materials and methods

### 2.1 Plant materials and growth conditions

Different lines of *Arabidopsis thaliana* seeds were used: wild type (WT, ecotype Columbia-0) and transgenic lines. The reporter line *ProNRT2.4:GFP* was in the Col-0 background (Kiba et al., 2012). The knockout (ko) mutant line *nrt2.4-1* corresponds to a T-DNA insertion in the last exon of the *NRT2.4* gene (MDL-ArBrAr-125) (Forsbach et al., 2003; Kiba et al., 2012). The knockout *nrt2.4-2* line was obtained from the Syngenta Arabidopsis Insertion Library (SAIL) T-DNA insertion line collection (SAIL_205_F02, stock CS872100). T-DNA insertion occurred in the third exon (Alonso et al., 2003; Kiba et al., 2012). No expression of *NRT2.4* could be detected by RT-PCR for either mutant line (Kiba et al., 2012). Genotyping using primers shown in **Supplementary Table S2**, confirmed T-DNA insertions in NRT2.4 gene in *nrt2.4-2* line, while the NRT2.5 gene was not disrupted.

*A. thaliana* seeds were surface sterilized using 25% (v/v) sodium hypochloride (ACROS Organics™, Germany) and 0.1% of Triton X-100 (Sigma-Aldrich, Germany) for 8 min, rinsed seven times with sterile water and grown on square plates (120×120×16mm) (Thermo Fisher Scientific, Germany) (12-15 seedlings per plate) containing N-complete (7 mM NO_3_^−^) MGRL medium (**Supplementary Table S1**). Seeds were stratified for 48 h at 4°C. According to Svietlova et al. (2023), plants are incubated for 14 days in a growth chamber in vertical position under long-day conditions (16 h light/8 h dark) and light intensity 100 μmol photos m^-2^ s^-1^, at 22°C. For the different N-source treatments (NO_3_^−^ or Gln), *A. thaliana* seedlings (6 per plate) were transferred for 10 d to MGRL medium (including 1% Sucrose, 0.5% Gelrite, pH 5.8) plates. Plates were either N-free (0 mM NO_3_^−^/0 mM Gln), N-low (0.25 mM NO_3_^−^/0.125 mM Gln) or N-complete (7 mM NO_3_^−^/3.5 mM Gln) supplemented with KCl and CaCl_2_·2H_2_O in appropriate quantity for supporting ion balance (**Supplementary Table S1**). Plants were harvested by 3 in each vial (roots and shoots separately) and weighed. At least 18 seedlings from each treatment were taken. The samples were frozen immediately in liquid N, and stored at −80°C for RNA preparation and amino acids analysis.

### 2.2 RNA preparation and expression analysis

Total RNA (2.5 µg) was extracted using TRIzol, according to the manufacturer’s method, followed by additional chloroform isolation and isopropanol precipitation steps; it was further digested from DNA contamination by TURBO *DNA-free™ KIT* (Life Technologies, USA) and cleaned by *RNA Clean and Concentrator™* KIT (™Trademarks of Zymo Research Corporation). The cDNA (20 µL) was synthesized using *Thermo Scientific RevertAid First Strand cDNA Synthesis KIT* (Thermo Fisher Scientific, Germany), according to the manufacturers’ instructions. The qPCR analysis was performed using Bio-Rad CFX96™ Real-Time System (Bio-Rad Laboratories Inc., USA) using the appropriate pairs for *A. thaliana* specific primers (**Supplementary Table S2**). The reaction components per 20 µL were as follows: 6.5 µL H_2_O, 12.5 µL Brilliant II SYBR Green qPCR Master Mix (Agilent Technologies, USA), 1 µL 10 µM of each primer and 1 µL cDNA. Thermal cycling program was as follows: initial denaturation at 95°C for 180 s, and 44 cycles at 95°C for 30 s, 60°C for 30 s, and 72°C for 30 s. *At*Actin II (AT3G18780) was used as an internal reference gene. The relative quantification of gene expression was evaluated using the delta-delta-Ct method according to Pfaffl (2001). Three biological replicates and three technical replicates were performed for each analysis.

### 2.3 Extraction and quantification of amino acids and abscisic acid by LC-MS/MS

The plant material was homogenized in a Geno/Grinder^®^ 2010 (Spex Sample Prep, Stanmore, UK) equipped with aluminum racks. Racks were cooled in liquid nitrogen before used to prevent thawing of the plant material throughout the homogenization process. The amino acids were extracted twice with a total of 2 mL of methanol on ice. Supernatants were combined and dried using a Concentrator plus (Eppendorf, Hamburg, Germany) and re-suspended in 500 μL of methanol. The extract was diluted 1:10 (v/v) with water containing the ^13^C, ^15^N-labeled amino acid mix (Isotec, Miamisburg, OH, USA) as the internal standard. Amino acids in the diluted extracts were directly analyzed by LC-MS/MS according to (Crocoll et al., 2016) with a QTRAP6500 mass spectrometer (Sciex, Darmstadt, Germany) coupled to an Agilent 1260 series HPLC system. The mass spectrometer was operated in positive ionization mode in multiple reaction monitoring mode (**Supplementary Table S3**). All amino acids were quantified relative to the peak area of the corresponding labeled compound, except for asparagine (using aspartate and a response factor of 1.0). Abscisic acid determination was carried out as described (Svietlova et al., 2023).

### 2.4 Analysis of gene expression in GFP reporter lines

Fluorescence microscopy of GFP signals was optimized for live cell and detected in roots during 10 days every 24 h after transfer the plants on N-depleted medium (Svietlova et al., 2023). Images were acquired using Zeiss AXIO Zoom.V16 (ZEISS, Germany, Oberkochen) equipped with 0.5× PlanApoZ Objective (ZEISS, Germany, Oberkochen), an HXP 120 mercury vapor lamp and a filter set 38 HE (excitation filter BP 450-490nm, FT 495nm, emission filter BP 500-550nm) for the visualization of GFP. Signal intensities after treatment were measured using Fiji ImageJ-2.9.0 Analysis Software. Images were converted to 8-bit and processed using Fiji’s “analyze particles” plugin. The average fluorescence intensity was measured in the cells of the apical lateral roots. For the measurement, ten randomly selected fluorescent points in the form of a square of four pixels for each plant were used. Fluorescence images were captured using a TOMOCUBE HT-X1 (Tomocube Inc., Republic of Korea) on 6th day of N starvation. HT-X1 model includes a 470 nm LED source, which was used to acquire 3D fluorescence images of GFP.

### 2.5 Statistical analysis

Independent experiments were treated as a completely randomized design. Figures were plotted using *GraphPad Prism* Software, version 9.0. Datasets of amino acids analysis was subjected using R studio version 1.1.463 with R version 3.4.4. (R Core Team, 2018). Statistically significant differences were calculated using One- and Two-Way-Analysis of Variance, with Dunnett’s multiple comparisons test with P<0.05 as the threshold for significance. Data were transformed if assumptions for statistical tests were not met.

## 3 Results

**Figure 1:**
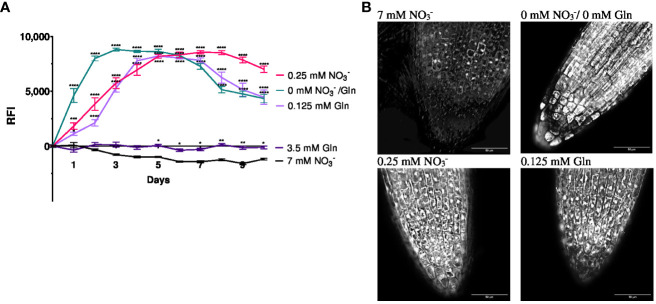
Relative fluorescence intensity (RFI) **(A)** and fluorescence microscopy **(B)** of *Arabidopsis thaliana* roots expressing the GFP reporter gene under control of *NRT2.4* promotor *(ProNRT2.4:GFP*). **(A)** Seedlings were pre-grown on full NO_3_^−^ (7 mM NO_3_^−^) medium. After two weeks, they grew for additional 10 d on different N-media: N-free (0 mM NO_3_^−^/0 mM Gln), N-low (0.25 mM NO_3_^−^/0.125 mM Gln) and N-complete (7mM NO_3_^−^/3.5 mM Gln) for the indicated time. Shown are the mean (n = 6–8); error bars indicate standard error (SEM). Statistical analysis was performed using repeated measures two-way ANOVA (F_Days_ = 156.7, F_Media_ = 674.0, P<0.0001) with Dunnett’s multiple comparisons test (each value compared to the respective 7 mM NO_3_^-^ value); *P<0.05; **P<0.01; *** P<0.001; **** P<0.0001; ns: not significant. **(B)** Fluorescence intensity of lateral roots treated with different N-sources. Results obtained after 6 d of N starvation using TOMOCUBE HT-X1 microscope.

**Figure 2:**
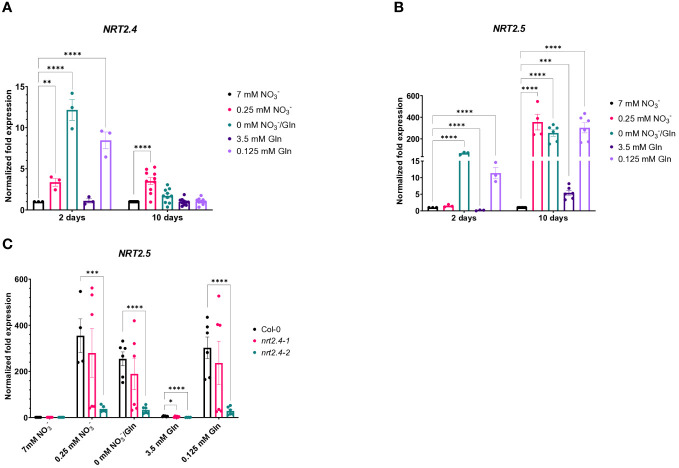
Quantitative PCR-based analysis of *NRT2.4* and *NRT2.5* expression in roots of *Arabidopsis thaliana* WT. Seedlings were pre-grown on full NO_3_^−^ (7 mM NO_3_^−^) medium. After two weeks, they grew for additional 10 d on different N-media: N-free (0 mM NO_3_^−^/0 mM Gln), N-low (0.25 mM NO_3_^−^/0.125 mM Gln) and N-complete (7mM NO_3_^−^/3.5 mM Gln). Expression of **(A)** *NRT2.4* and **(B)** *NRT2.5* on day 2 and 10 after transfer in Col-0 WT. **(C)** Expression of *NRT2.5* in WT and *nrt2.4* ko mutants 10 d after transfer. Shown are the mean (n = 3–10); error bars indicate standard error (SEM). Statistical analysis was performed using Two-way ANOVA (A: F_Days_ = 47.35, F_Media_ = 22.76, P<0.0001; B: F_Days_ =219.0, F_Media_ = 128.7, P<0.0001; C: F_Media_ = 158.0, F_Lines_ = 37.1, P<0.0001) with Dunnett’s multiple comparisons test (each value compared to the respective control value); **P<0.01; *** P<0.001; **** P<0.0001.

In order to study if NRT2.4 as part of the high affinity nitrate uptake system is affected by different N sources (NO_3_^−^ or Gln), the well described *ProNRT2.4:GFP* reporter line (Kiba et al., 2012) was employed (**Figure 1**). While fluorescence was detectable immediately and reached a maximum 2 d after transfer to no N conditions, at low N the fluorescence enhancement was also detectable although slightly slower and reached maxima after 4 d, independent on the N source, NO_3_^−^ or Gln. No induction occurred on both full N media, neither with 7 mM NO_3_^−^ nor with 3.5 mM Gln (**Figure 1A**). TOMOCUBE HT-X1 microscopy of lateral Arabidopsis roots, which were treated with different N-sources, supported these results (**Figure 1B**). Moreover, a corresponding qPCR analysis confirmed the rapid *NRT2.4* gene induction over time under N-deficiency in Arabidopsis WT roots (**Figure 2A**). At day 2, the increase in NRT2.4 transcripts was 12.2-fold under N-free conditions, 8.5-fold at 0.125 mM Gln, and 3.4-fold at 0.25 mM NO_3_^−^, all compared to controls grown with 7 mM nitrate. At day 10, almost no *NRT2.4* gene induction was detectable at the different N-concentrations, only a remaining 3.5-fold increase was found on 0.25 mM NO_3_^−^, suggesting an early but transient induction of this transporter (**Figure 2A**).

Given that the high affinity nitrate transporter NRT2.5 is induced the most among the seven NRT2 family members in Arabidopsis under long-term nitrogen starvation, and *NRT2.5* becomes the most abundant transcript (Lezhneva et al., 2014), we also investigated the expression of *NRT2.5* upon N-depletion compared to controls grown with 7 mM nitrate (**Figure 2B**). In contrast to *NRT2.4*, *NRT2.5* expression was relatively lower at day 2 in comparison to day 10. While at day 2 only under no N or 0.125 mM Gln *NRT2.5* expression was detectable, at day 10 its expression was obvious, raising from only 5.4-fold on 3.5 mM Gln up to 355-fold on low NO_3_^−^, respectively (**Figure 2B**). No or very low expression of these two genes (*NRT2.4* and *NRT2.5*) belonging to the HATS family was found upon full Gln treatment.

**Figure 3:**
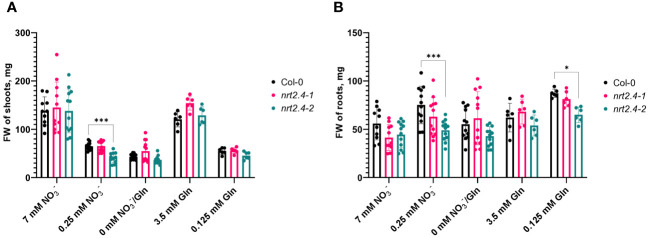
Fresh weight of *Arabidopsis thaliana* WT and *nrt2.4* ko mutant plants during N starvation. Shoots **(A)** and roots **(B)**. Seedlings were pre-grown on full NO_3_^-^ (7 mM NO_3_^−^) medium. After two weeks, they grew for additional 10 d on different N-media: N-free (0 mM NO_3_^−^/0 mM Gln), N-low (0.25 mM NO_3_^−^/0.125 mM Gln) and N-complete (7mM NO_3_^−^/3.5 mM Gln). Shown are the mean (n = 6–12); error bars indicate standard deviation (SD). Statistical analysis was performed using Two-way ANOVA (A: F_media_ = 152.6, F_Lines_ = 12.6, P<0.0001; B: F_Media_ = 12.6, F_Lines_ = 11.9, P<0.0001) with Dunnett’s multiple comparisons test (each value compared to the respective Col-0 value); *P<0.05; *** P<0.001.

Since Kiba et al. (2012) found a decreased NO_3_^−^ uptake in *nrt2.4* ko mutants under N-starvation conditions (up to 30% less uptake was observed at both 0.025 and 0.01 mM external NO_3_^−^), we also included the two Arabidopsis ko mutant lines (*nrt2.4-1* and *nrt2.4-2)* in the analysis. An unexpected finding was that *NRT2.5* expression in *nrt2.4-2* ko plants was drastically reduced in comparison to WT or *nrt2.4-1* ko plants under all N-depletion conditions (**Figure 2C**). This result was reflected in growth of *nrt2.4-2* ko plants (**Figure 3**). The fresh weight of both shoots (**Figure 3A**) and roots (**Figure 3B**) was slightly but significantly reduced in *nrt2.4-2* plants under low N-conditions compared to WT or *nrt2.4-1* plants.

**Figure 4:**
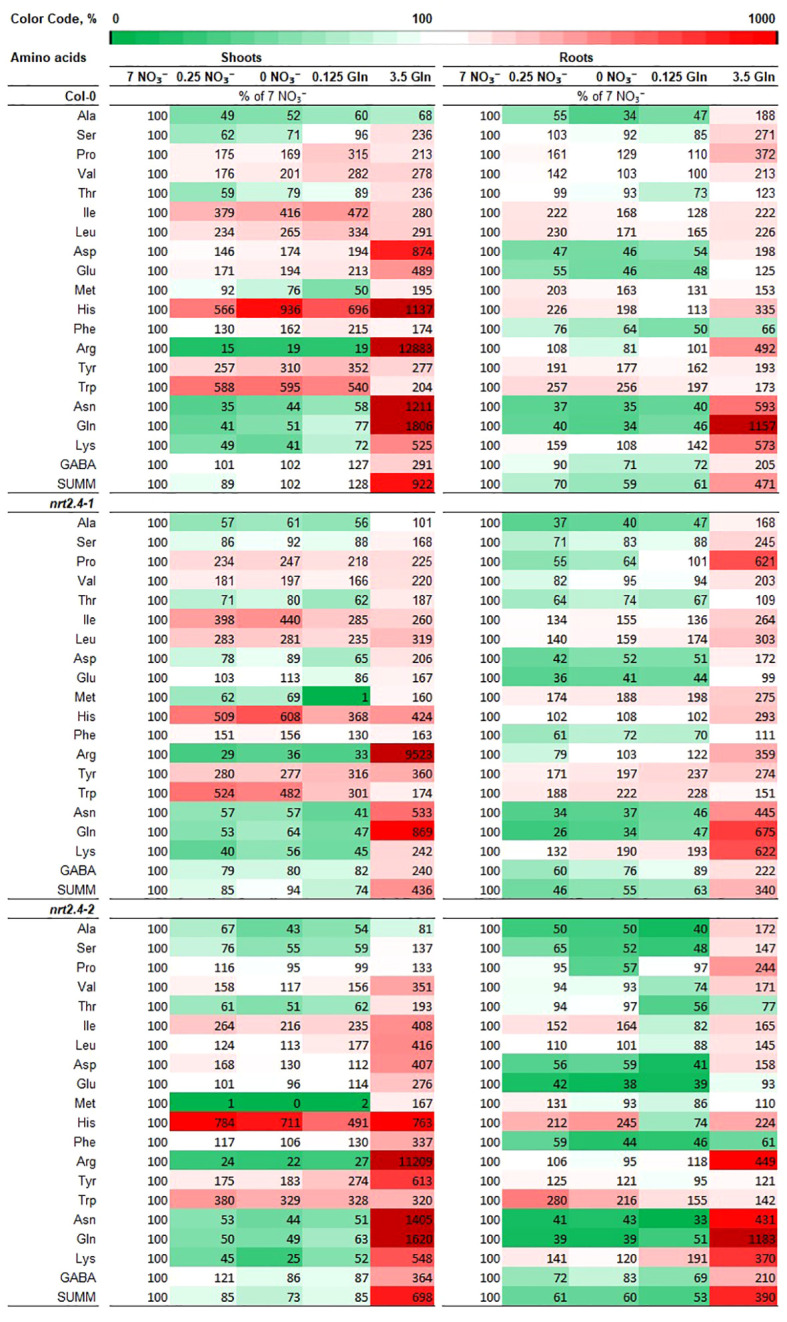
Heat map of free amino acids levels in shoots and roots in *Arabidopsis thaliana* WT and *nrt2.4* ko mutants during N starvation. Seedlings were pre-grown on full NO_3_^-^ (7 mM NO_3_^−^) medium. After two weeks, they grew for additional 10 d on different N-media: N-free (0 mM NO_3_^−^/0 mM Gln), N-low (0.25 mM NO_3_^−^/0.125 mM Gln) and N-complete (7mM NO_3_^−^/3.5 mM Gln). Amino acid profiles were identified 10 d after transfer. Data are given as the percentage of full NO_3_^-^ (7 mM NO_3_^−^) medium; n = 3.

Considering that Gln is the first organic nitrogenous molecule formed from inorganic nitrogen and the precursor of all other amino acids (AA), the free AA content upon growth on the different Gln concentrations was studied in detail. Compositions and changes in the AA pools in the different Arabidopsis lines were analyzed individually in both shoots and roots, depending on the given N-level in the medium. Not surprisingly, we found significant differences in AA profiles of plants at different N-sources (**Figure 4**). The specificity of these changes is evident. While no obvious differences were found when nitrate or Gln were applied as N-source at low concentrations, a striking change in AAs was found when external Gln was applied at a concentration of 3.5 mM. This applies to both WT and ko mutant plants. Looking deeper in the AA results, it is interesting to note that in WT in both shoots and roots high exogenous Gln had a particular strong effect on the accumulation of AA containing two (Asn, Gln, Lys), three (His) or four (Arg) nitrogen atoms. These changes were more pronounced in shoots than roots. The same trend also occurred in shoots and roots of *nrt2.4-1* and *nrt2.4-2* ko mutant plants (**Figure 4**). A statistical analysis between the three different lines showed that in the root almost no differences were detected in contrast to shoots (**Supplementary Table S4**). Here, in *nrt2.4-2*, the content of six AAs (Val, Ile, Leu, Phe, Tyr, Trp) was statistically different to both the Col-0 WT and *nrt2.4-1* plants.

**Figure 5:**
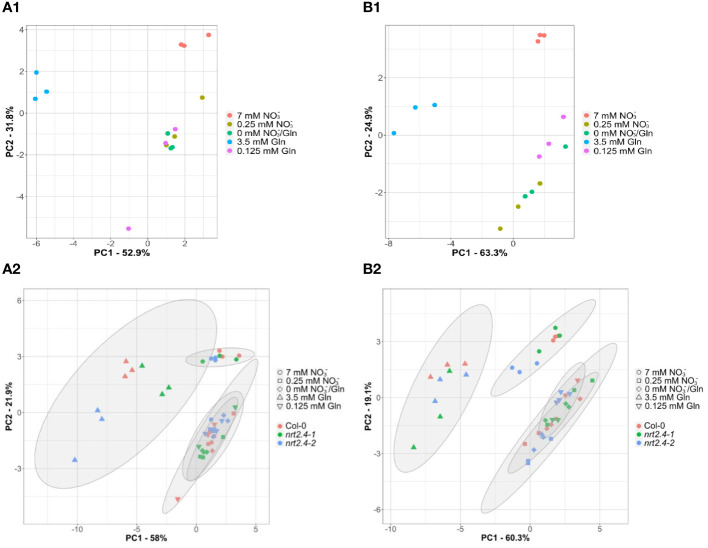
Principal component analyses (PCA) of free amino acid compositions in *Arabidopsis thaliana* shoots **(A)** and roots **(B)** during N starvation. The PCA score plot distinguishes the amino acid profiles of WT plants **(A1, B1)** or WT and mutant lines (Col-0; *nrt2.4-1; nrt2.4-2*) **(A2, B2)** grown under different treatments of N-starvation: N-free (0 mM NO_3_^−^/0 mM Gln), N-low (0.25 mM NO_3_^−^/0.125 mM Gln) and N-complete (7mM NO_3_^−^/3.5 mM Gln). **(A2, B2)** Amino acid profiles were separately analyzed after 10 d. The ellipses represent the multivariate normal distribution.

For Arabidopsis, Huang and Jander (2017) have shown that nutrient deficiency can lead to ABA-regulated protein degradation. To get an idea of the origin of the AAs that increased under our experimental conditions, we analyzed the ABA contents in the different lines. Almost no changes in ABA content were observed in roots (**Supplementary Figure S1A**). In contrast, a clear increase in ABA content was observed in shoots growing on media with lower nitrogen concentrations compared to the 7 mM nitrate control (**Figure S1B**). While this can be described as a clear trend for the Col-0 and *nrt2.4-1* line, the differences in *nrt2.4-2* were all significant. For further and deeper statistical analysis, we performed a principle component analysis (PCA, **Figure 5**) of AA compositions. This revealed clear separation of three clusters; i.e. between both full media (7 mM NO_3_^−^, 3.5 Gln), and all low and no nitrate media (0.25 mM NO_3_^−^, 0.125 mM Gln and 0 mM NO_3_^−^/0 mM Gln) in shoots (**Figure 5A1**) and in roots (**Figure 5B1**) of WT plants. The same cluster separation was obtained in an analysis where WT and the two mutant lines were included (**Figures 5A2, B2**). In any case, confidence areas of no and low N overlap sufficiently in shoots and roots. The two principal components, PC1 and PC2, explain in both shoots and roots about 80% or more of all observed variances.

## 4 Discussion

The decrease in NO_3_^−^ uptake by the roots in plants with sufficient nitrogen levels is caused by feedback regulation from the end-products of NO_3_^−^ assimilation such as amino acids. For example, Thornton (2004) reported that the maximal influx rate associated with HATS was reduced by 66% in the presence of Gln in *Lolium perenne* plants, while LATS-associated influx remained unaffected. Other studies with different nitrogen supply systems showed both the induction of HATS - such as NRT2 activities - by NO_3_^−^ deprivation and the suppression or reduction of HATS induction by the simultaneous or alternative supply of AAs (Zhuo et al., 1999; Nazoa et al., 2003; Thornton, 2004; Miller et al., 2008; Zoufan and Shariati, 2009). Amino acid analysis showed that this repression was specifically related to enhanced internal level of Gln, suggesting a particular role for this amino acid in nitrogen signaling in general, including nitrate uptake regulation (Miller et al., 2008; Lee et al., 2023). These results were obtained with exogenous application of Gln in combination with different concentrations of NO_3_^−^ as background, representing a combination of the two N-sources, whereby additional Gln always alleviated plant nitrogen deficiency. Stimulated by these data, we aimed here to investigate the effects of exogenous Gln as the sole source of nitrogen on HATS, in particular on *NRT2.4* and *NRT2.5*, in Arabidopsis.

Nitrogen starvation-induced *NRT2.4* expression is known to decrease steadily with increasing NO_3_^−^ concentration in the medium (98% decrease between 0 and 10 mM NO_3_^−^). In addition, low expression of the *NRT2.4* gene can also occur when NH_4_^+^ is present in the media (Kiba et al., 2012) and Gln has been described as a signaling molecule to regulate gene expression in plants (Kan et al., 2015). Therefore, we investigated whether or not *NRT2.4* expression was affected by an organic N-source, Gln, in comparison with NO_3_^−^. Using an Arabidopsis reporter line expressing GFP under the control of the *NRT2.4* promotor (*ProNRT2.4:GFP*; Kiba et al., 2012), the GFP expression was faster under no nitrate and no expression was detectable under full N-supply, independent of the form (3.5 mM Gln or 7 mM NO_3_^−^) (**Figure 1A**). This finding supports results from Kiba et al. (2012) who showed no induction of *NRT2.4* expression upon transfer of Arabidopsis plants from a NO_3_^−^-containing medium to a medium with NH_4_^+^ as nitrogen source. Surprisingly, in a recent study Chaput et al. (2023) found NRT2.4 induction after such a transfer on NH_4_^+^-medium. Strikingly, the GFP expression was very similar when the plants were supplied with 0.25 mM NO_3_^−^ or 0.125 mM Gln, respectively, providing the same amount of N-atoms (**Figure 1**). These results provide further evidence that the N-sensor responsible for HATS induction is neither their substrate, NO_3_^−^, nor any other inorganic N-containing compound. More likely, the internal pools of amino acids might indicate the nitrogen status by providing a signal that can regulate NO_3_^−^ uptake by the plant. The regulation of HATS expression thus shows a certain non-specificity and dependence on the general content or organic nitrogen and not on the NO_3_^−^ content.

Our data in addition showed a low level of *NRT2.4* transcript expression, which varied between a 12.2-fold increase in N-free medium after 2 days and a 3.5-fold increase in 0.25 mM NO_3_^−^ after 10 days (**Figure 2A**). While under no nitrate conditions, *NRT2.4* gene was transiently but already highly expressed after 2 d (**Figure 2A**), *NRT2.5* was induced as well but with a different kinetics, i.e. much higher after 10 d than after 2 d of nitrate deficiency (**Figure 2B**). Moreover, in contrast to *NRT2.4, NRT2.5* transcript expression was *per se* higher, showing a 5.5-fold increase on full Gln source and 355-fold increase on 0.25 mM NO_3_^−^ after 10 days, compared with 7 mM NO_3_^−^ (**Figure 2B**), supporting results by Lezhneva et al. (2014). Such expression of *NRT2.5* may explain why *nrt2.4* ko mutant plant can survive even under strong N-deficiency, suggesting that the missing *NRT2.4* could be compensated by other nitrate uptake systems and the mutant lines show similar growth performance (**Figure 3**). Analysis of *NRT2.5* gene expression in the *nrt2.4* ko-mutants supported this hypothesis, at least for *nrt2.4-1* (**Figure 2C**). However, we found a difference in *NRT2.5* transcript expression in the two *nrt2.4* ko-mutants: high *NRT2.5* expression in *nrt2.4-1* and a strongly reduced expression in *nrt2.4-2* (**Figure 2C**). It is tempting to speculate that the even further impaired N-supply in the *nrt2.4-2* mutant line could explain the particular difference in growth under low or no N-sources compared to *nrt2.4-1*. This can be seen in **Figure 3** in the fresh weight of roots and shoots after 10 days of N deprivation.

Low exogenous Gln concentration (0.125 mM) as the only N-supply had similar effects on the AA pools as no or 0.25 mM NO_3_^−^ (**Figures 4**, **5**). As indicate by PCA, only the full N-sources (3.5 mM Gln, 7 mM NO_3_^−^) clustered differently, even from each other (**Figure 5**). This strongly suggests that low Gln concentrations have similar effects as comparable NO_3_^−^ concentrations in contrast to higher Gln concentrations. Once the source of organic bound nitrogen is very high, the plants seemingly fill nitrogen stores. A particular strong and different effect on the AA pools was detected in WT and *nrt2.4* ko plants upon high Gln supply in both roots and even more pronounced in shoots (**Figure 4**; **Supplementary Table S4**). High exogenous Gln significantly increased the level of AAs containing two (Asn, Gln, Lys, Trp), three (His) or four (Arg) nitrogen in chemical structure in both shoots and roots suggesting a role of AA as N storage compounds (**Figure 4**). In particular Arg is also a precursor for the biosynthesis of oxidative stress-related NO production as well as for polyamines such as spermine, spermidine and putrescine. Various studies have demonstrated that polyamines are required for plant growth and development (Kawade et al., 2023).

Gln is the first nitrogen-containing organic compound, which is involved via transamination to generate other AAs. Root-to-shoot movement of AAs occurs in the xylem and xylem loading with Gln is known for a long time (Schobert and Komor, 1990). Gln and Arg are most abundant in the xylem sap, whereas all amino acids are transported through the phloem (Yao et al., 2020). Here, AA transporters of the AAP or LHT types might be involved (Guo et al., 2021). Another observation was the increase of minor AAs (e.g. Leu, Ile, Val, Pro, Tyr, Trp) when plant grew at low nitrogen (**Figure 4**). Not at least here, the question for the origin of these AAs raised, *de novo* or from protein degradation (Rhodes et al., 1986, Rhodes et al., 1987). A study of Huang and Jander (2017) demonstrated that in Arabidopsis abiotic stress, including nutrient deficiency was able to induce protein degradation and subsequently the accumulation of free AAs, in particular branched-chain amino acids (BCAAs). This protein degradation was depending on ABA. Moreover, it is suggested that nutrients such as nitrogen may function via a TOR-based pathway (Shi et al., 2018). For example, *Arabidopsis* seedlings overexpressing TOR are hyposensitive to high nitrate inhibition of roots. AAs may also activate TOR signaling pathways (Shi et al., 2018). Strikingly, ABA can repress TOR signaling by activation of SnRK2s, plant-specific serine/threonine kinases involved in response to abiotic stresses (Wang et al., 2018). Due to such findings, we chose an indirect approach and analyzed the ABA content in the different Arabidopsis lines and different nitrogen supply approaches (**Figure S1**). While in roots only minor changes in ABA contents were detectable, there was a clear increase of ABA in shoots upon growth on low nitrogen sources (**Supplementary Figure S1B**). These results suggest at least the involvement of protein degradation in shoots in particular in *nrt2.4-2* and supported by our finding of increase of BCAAs (Val, Ile, Leu) as well as of aromatic AAs (Tyr, Phe, Trp) (**Figure 4**; **Supplementary Table S4**). Huang and Jander (2017) have found similar results upon nutrient deficiency. In order to find a clear explanation for the origin of the AAs that increased, more experiments with ^15^N-labelled precursors of AAs synthesis should be performed (Rhodes et al., 1986; Rhodes et al., 1987). In addition, a connection between nitrogen deficiency, ABA and AAs increase, and TOR signaling is conceivable and needs further studies.

The reasons for the unexpected results concerning the affected *NRT2.5* expression in *nrt2.4-2* are not clear. From Kiba et al. (2012), it is known that both ko lines lack the *NRT2.4* transcript in RT-PCR. However, it is conceivable that due to the T-DNA insertion an unknown truncated protein is produced, which somehow affects *NRT2.5* gene expression. This could be a direct physical interaction as well as disturbance of regulatory processes. Since *NRT2.5* expression can be detected in *nrt2.4-1* plants but not in *nrt2.4-2*, it is more likely that only this latter mutant line has a side or off-target effect. Obviously, the additional effect on *NRT2.5* has more consequences for this particular plant line. Beside a slightly reduced growth (**Figure 3**) the content of various AAs in shoots is significantly different compared to WT and *nrt2.4-1* plants (**Supplementary Table S4**). For all these AAs, a higher level was detected in *nrt2.4-2* suggesting an impact on AA metabolism. This seems to be restricted to the shoots. Overall, but unfortunately beyond the scope of this study, it is necessary to find out the differences between the *nrt2.4-1* and *nrt2.4-2* mutant lines at the molecular level that cause their different nature.

## Data availability statement

The original contributions presented in the study are included in the article/**Supplementary Material**. Further inquiries can be directed to the corresponding author.

## Author contributions

NS: Conceptualization, Investigation, Methodology, Writing – original draft, Writing – review & editing. LZ: Formal analysis, Software, Visualization, Writing – review & editing. MR: Investigation, Writing – review & editing. VG: Data curation, Resources, Writing – review & editing. AM: Conceptualization, Funding acquisition, Supervision, Writing – original draft, Writing – review & editing.
