# Supplementary material for: Glutamine as sole nitrogen source prevents induction of nitrate transporter gene NRT2.4 and affects amino acid metabolism in Arabidopsis
Source: Front Plant Sci. 2024 Mar 25;15:1369543. doi: 10.3389/fpls.2024.1369543 (PMC11022244; doi:10.3389/fpls.2024.1369543)
Supplement: Supplementary file 1 [file DataSheet_1.docx]

Glutamine as Sole Nitrogen Source Prevents Induction of Nitrate Transporter gene *NRT2.4* and Affects Amino Acid Metabolism in Arabidopsis

Nataliia Svietlova^1^*,* Liza Zhyr^1^, Michael Reichelt^2^, Veit Grabe^3^*,* Axel Mithöfer^1^*

Supplementary Material

**Supplementary Table S1.** MGRL medium composition.

| **Nutrients/Chemicals** | **MGRL/7mM NO_3_^-^** | **MGRL/0.25 mM NO_3_^-^** | **MGRL/0 mM NO_3_^-^** | **MGRL/0.125 mM Gln** | **MGRL/3.5 mM Gln** |
| --- | --- | --- | --- | --- | --- |
| KNO_3_ | 3 mM | 0.11 mM | NA | NA | NA |
| Ca(NO_3_)_2_·5H_2_O | 2 mM | 0.07 mM | NA | NA | NA |
| KCl | NA | 2.89 mM | 3 mM | 2.96 mM | NA |
| CaCl_2_·2H_2_O | NA | 1.93 mM | 2 mM | 1.96 mM | NA |
| Na_2_HPO_4_·7H_2_O (pH=5.8) | 85.3 µM | 85.3 µM | 85.3 µM | 85.3 µM | 85.3 µM |
| NaH_2_PO_4_·H_2_O (pH=5.8) | 1.67 mM | 1.67 mM | 1.67 mM | 1.67 mM | 1.67 mM |
| MgSO_4_·7H_2_O | 1.5 mM | 1.5 mM | 1.5 mM | 1.5 mM | 1.5 mM |
| Na_2_‐EDTA·2H_2_O | 67 µM | 67 µM | 67 µM | 67 µM | 67 µM |
| H_3_BO_3_ | 30 µM | 30 µM | 30 µM | 30 µM | 30 µM |
| MnSO_4_·7H_2_O | 10.3 µM | 10.3 µM | 10.3 µM | 10.3 µM | 10.3 µM |
| FeSO_4_·7H_2_O | 8.6 µM | 8.6 µM | 8.6 µM | 8.6 µM | 8.6 µM |
| CuSO_4_·5H_2_O | 1 µM | 1 µM | 1 µM | 1 µM | 1 µM |
| CoCl2·6H2O | 130 nM | 130 nM | 130 nM | 130 nM | 130 nM |
| (NH_4_)_6_Mo_7_O_24_·4H_2_O | 24 nM | 24 nM | 24 nM | 24 nM | 24 nM |
| ZnSO_4_·7H_2_O | 1 µM | 1 µM | 1 µM | 1 µM | 1 µM |
| L-Glutamine | NA | NA | NA | 0.125 mM | 3.5 mM |
| Sucrose | 1.0% w/v | 1.0% w/v | 1.0% w/v | 1.0% w/v | 1.0% w/v |
| Gelrite™ | 0.5% w/v | 0.5% w/v | 0.5% w/v | 0.5% w/v | 0.5% w/v |

**Supplementary Table S2.** Oligonucleotides used for quantitative RT-PCR (**A**) and *nrt2.4-2* line genotyping (**B**).

**A**

| **Primer name** | **Sequence 5’→ 3’** | **Target** |
| --- | --- | --- |
| AtNRT2.4 forward | CAGTTCCTTCCGACTCATCA | AT5G60770 |
| AtNRT2.4 reverse | GCAACACCAGCATTTCCGAC | AT5G60770 |
| AtNRT2.5 forward | CTCCTCCCTGTTATCCGTGAAA | AT1G12940 |
| AtNRT2.5 reverse | AGACGAAAGTGGCGAGAGAGAA | AT1G12940 |
| AtActin II forward | GGAATCCACGAGACAACCTA | AT3G18780 |
| AtActin II reverse | ATCTTCATGCTGCTTGGTGC | AT3G18780 |

**B**

| **Mutant line** | **Primer name** | **Sequence 5’→ 3’** | **Target** |
| --- | --- | --- | --- |
| SAIL_205_F02 | LP2 | TAGGGTCTGCAGATCGTTGTC | AT5G60770 |
|  | RP2 | CACCAGCTCCAGTAAGACCAG | AT5G60770 |
|  | LB1 | GCCTTTTCAGAAATGGATAAATAGCCTTGCTTCC | T-DNA-specific primer |
| Gabi_213H10 | LP | GATGAGCTCCATGTTCTCTGG | AT1G12940 |
|  | RP | ATCAACTGTGTTAAGACCGCG | AT1G12940 |
|  | o8474 | ATAATAACGCTGCGGACATCTACATTTT | T-DNA-specific primer |

**Supplementary Table S3.** Details of analysis of amino acids by LC-MS/MS [HPLC 1260 (Agilent Technologies)-QTRAP6500 (AB SCIEX)] in positive ionization mode.

| **Compound** | **Q1** | **Q3** | **RT (min)** | **Internal**  **standard** | **IS**  **Q1** | **IS**  **Q3** | **DP** | **CE** |
| --- | --- | --- | --- | --- | --- | --- | --- | --- |
| Ala | 90.1 | 44.1 | 0.5 | 13C,15N-Ala | 94.1 | 47.1 | 20 | 17 |
| Ser | 106.0 | 60.1 | 0.5 | 13C,15N-Ser | 110.0 | 63.1 | 20 | 15 |
| Pro | 116.1 | 70 | 0.5 | 13C,15N-Pro | 122.1 | 75.0 | 20 | 19 |
| Val | 118.1 | 72.2 | 0.5 | 13C,15N-Val | 124.1 | 77.2 | 20 | 13 |
| Thr | 120.1 | 74.2 | 0.5 | 13C,15N-Thr | 125.1 | 78.2 | 20 | 13 |
| Ile | 132.2 | 86.1 | 1.1 | 13C,15N-Ile | 139.2 | 92.1 | 20 | 13 |
| Leu | 132.2 | 86.1 | 1.3 | 13C,15N-Leu | 139.2 | 92.1 | 20 | 13 |
| Asp | 134.1 | 74.1 | 0.5 | 13C,15N-Asp | 139.1 | 77.1 | 20 | 19 |
| Glu | 148.1 | 102.1 | 0.5 | 13C,15N-Glu | 154.1 | 107.1 | 20 | 15 |
| Met | 150.2 | 104.1 | 0.7 | 13C,15N-Met | 156.2 | 109.1 | 20 | 13 |
| His | 156.2 | 110.1 | 0.4 | 13C,15N-His | 165.2 | 118.1 | 20 | 17 |
| Phe | 166.2 | 120.2 | 2.6 | 13C,15N-Phe | 176.2 | 129.2 | 20 | 17 |
| Arg | 175.1 | 70.1 | 0.4 | 13C,15N-Arg | 185.1 | 75.1 | 20 | 31 |
| Tyr | 182.1 | 136.2 | 1.4 | 13C,15N-Tyr | 192.1 | 145.2 | 20 | 17 |
| Asn | 133.1 | 74.1 | 0.5 | 13C,15N-Asp |  |  | 20 | 21 |
| Gln | 147.1 | 130 | 0.5 | 13C,15N-Gln | 154.1 | 136.0 | 20 | 13 |
| Trp | 205.2 | 188.1 | 3.2 | D5-Trp | 210.0 | 193.0 | 20 | 13 |

**Supplementary Table S4. Results of statistical analysis of free amino acids accumulation in *A. thaliana* plant lines (Col-0, *nrt2.4-1* and *nrt2.4-2)* grown for 10 days on 3.5 mM Gln.**

| **Shoot - 3.5 mM Gln** | | | |
| --- | --- | --- | --- |
|  | ***Col-0 / nrt2.4-1*** | ***Col-0 / nrt2.4-2*** | ***nrt2.4-1/ nrt2.4-2*** |
| **Ala** | 0.050 | 0.464 | 0.244 |
| **Ser** | **0.002** | 0.081 | **0.037** |
| **Pro** | 0.842 | 0.112 | 0.231 |
| **Val** | 0.887 | **0.006** | **0.004** |
| **Thr** | 0.949 | 0.999 | 0.933 |
| **Ile** | 0.960 | **0.004** | **0.005** |
| **Leu** | 0.710 | **0.014** | **0.035** |
| **Asp** | **0.035** | 0.107 | 0.657 |
| **Glu** | 0.074 | 0.971 | 0.056 |
| **Met** | **0.010** | 0.751 | **0.005** |
| **His** | **0.043** | 0.788 | 0.100 |
| **Phe** | 0.989 | **0.003** | **0.003** |
| **Arg** | 0.065 | 0.668 | 0.199 |
| **Tyr** | 0.534 | **0.006** | **0.021** |
| **Trp** | 0.722 | **0.005** | **0.012** |
| **Asn** | 0.096 | 0.634 | **0.030** |
| **Gln** | 0.082 | 0.990 | 0.097 |
| **Lys** | 0.954 | 0.259 | 0.180 |
|  |  |  |  |
| **Root - 3.5 mM Gln** | | | |
|  | ***Col-0 / nrt2.4-1*** | ***Col-0 / nrt2.4-2*** | ***nrt2.4-1 / nrt2.4-2*** |
| **Ala** | 0.313 | 0.665 | 0.764 |
| **Ser** | 0.593 | 0.810 | 0.920 |
| **Pro** | **0.010** | 0.956 | **0.013** |
| **Val** | 0.245 | 0.669 | 0.646 |
| **Thr** | 0.771 | 0.596 | 0.275 |
| **Ile** | 0.343 | 0.721 | 0.751 |
| **Leu** | 0.208 | 0.684 | 0.558 |
| **Asp** | 0.053 | 0.316 | 0.389 |
| **Glu** | 0.178 | 0.992 | 0.206 |
| **Met** | 0.111 | 0.944 | 0.074 |
| **His** | 0.571 | 0.934 | 0.769 |
| **Phe** | 0.194 | 0.959 | 0.273 |
| **Arg** | 0.094 | 0.205 | 0.820 |
| **Tyr** | 0.271 | 0.367 | 0.965 |
| **Trp** | 0.972 | 0.057 | **0.043** |
| **Asn** | 0.099 | 0.419 | 0.512 |
| **Gln** | 0.452 | 0.988 | 0.383 |
| **Lys** | 0.693 | 0.464 | 0.908 |

One-Way ANOVA test performed on amino acid levels separately in shoots and roots. Multiple comparison was performed using TukeyHSD test. Statistically significant differences (P < 0.05) marked in red.


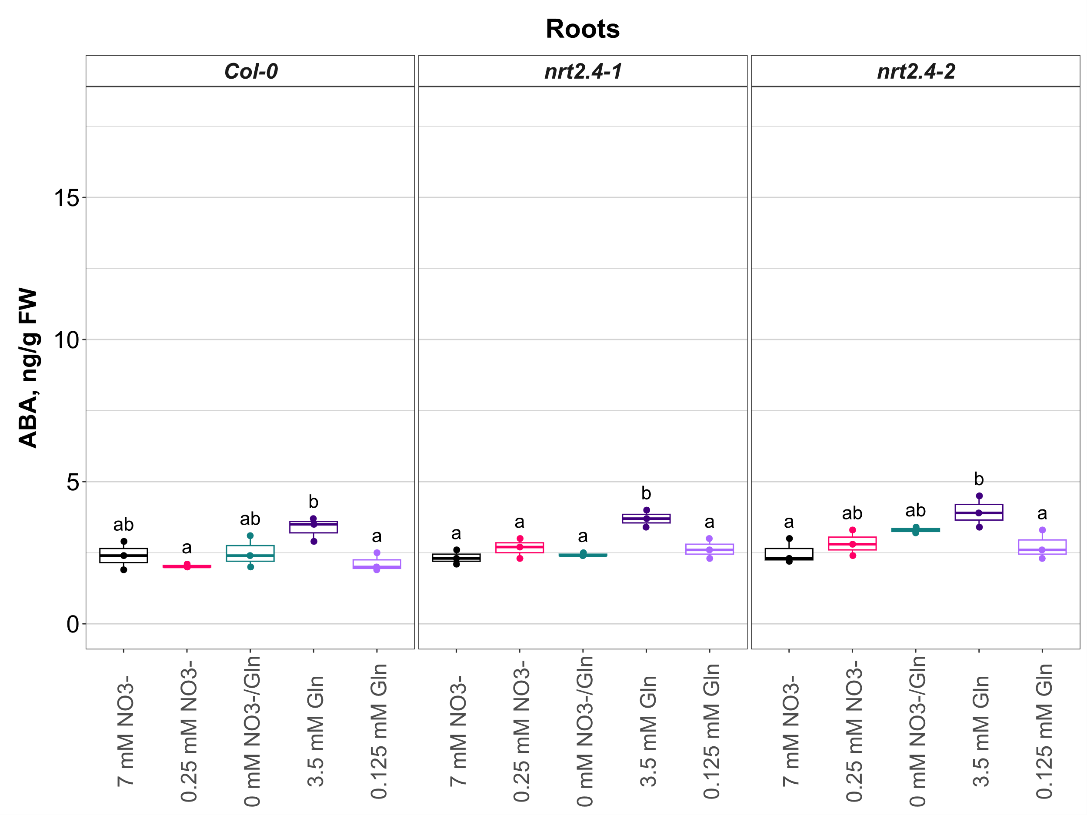


**A**

**B**


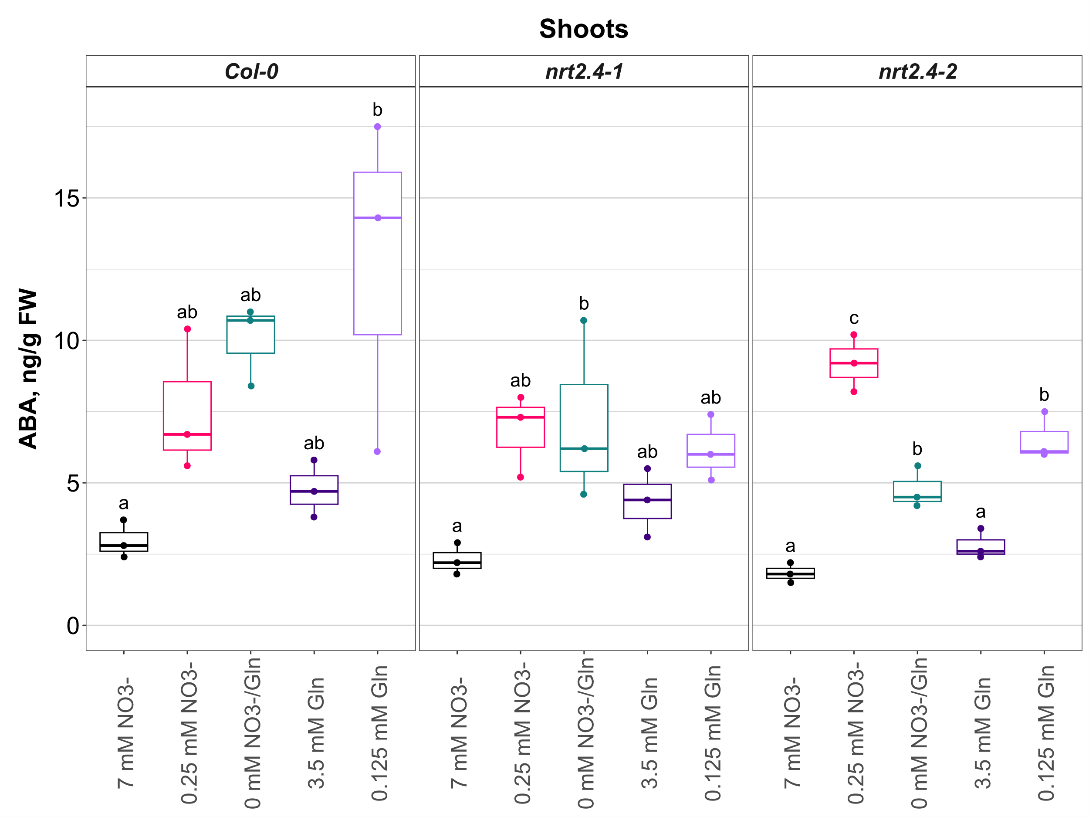


**Supplementary Figure S1**: Abscisic acid (ABA) concentrations in roots (A) and shoots (B) of *Arabidopsis thaliana* plants (Col-0, *nrt2.4-1, nrt2.4-2*). Seedlings were pre-grown on full NO_3_^−^ (7 mM NO_3_^−^) medium. After two weeks, they grew for additional 10 d on the indicated media. ABA was measured 10 d after transfer. Shown are the results of n = 3. Boxes are limited by lower and upper quartiles with a centre line representing the median; whisker length is restricted to 1.5 times the interquartile range; individual values are shown as dots. Different letters indicate significant differences (P<0.05) within one *A. thaliana* line based on one-way ANOVA (A: F_Col-0_ = 4.97, P_Col-0_ = 0.018, F_nrt2.4-1_ = 11.13, P_nrt2.4-1_ = 0.001, F_nrt2.4-2_ = 4.99, P_nrt2.4-2_ = 0.018; B: F_Col-0_ = 5.13, P_Col-0_ = 0.016, F_nrt2.4-1_ = 4.06, P_nrt2.4-1_ = 0.033, F_nrt2.4-2_ = 49.4, P_nrt2.4-2_ <0.0001) followed by TukeyHSD post-hoc test.
